# Cultural adaptation, translation and validation of the Spanish version of Past-day Adults’ Sedentary Time

**DOI:** 10.1186/s12889-021-10205-0

**Published:** 2021-01-21

**Authors:** Nicolas Aguilar-Farias, Pía Martino-Fuentealba, Damian Chandia-Poblete

**Affiliations:** 1 Department of Physical Education, Sports and Recreation. Universidad de La Frontera, Av Francisco Salazar 01145, 4780000 Temuco, Chile; 2 UFRO Activate Research Group, Universidad de La Frontera, Temuco, Chile; 3 School of Public Health and Social Work, Queensland University of Technology, Brisbane, Australia

**Keywords:** Sedentary behaviour, Sitting, Validity, Questionnaire, Self-report

## Abstract

**Background:**

To better understand sedentary behaviour and favour international comparisons, more evidence from different countries are needed. However, there are a few tools available in Spanish to measure sedentary behaviour. This study aimed to culturally adapt, translate and validate the Past-day Adults’ Sedentary Time (PAST) questionnaire in Chilean adults compared with the ActivPAL.

**Method:**

One hundred one workers wore an ActivPAL for 1 week and were asked to respond to the Spanish version of the PAST twice on different visits at a 7-day interval. The PAST assesses sedentary behaviour in several domains, including working time, during the previous day. Reliability was assessed with the intraclass correlation coefficient (ICC). Correlations and Bland-Altman methods were used to determine accuracy properties of the PAST compared with the ActivPAL.

**Results:**

Seventy-seven participants provided valid data (51.0% male; age = 39.0 ± 12.39 years). The PAST showed moderate reliability (ICC = 0.63). For the total time in sedentary behavior per day, the PAST showed no correlation (*r* = 0.21, *p* = 0.07) and a mean bias of 54.9 min/day (LoA 95%: − 484.3, 594.2 min/day) with the ActivPAL. For the total time in SB at work, the PAST showed moderate reliability (ICC = 0.40), weak correlation (*r* = 0.37, *p* < 0.002), and mean bias was 33.8 min/day (LoA 95%: − 285.7, 353.3 min/day).

**Conclusions:**

The PAST performed better when estimating sedentary behaviour during working hours compared with the whole day. In this setting, accuracy properties were comparable with other self-report tools.

## Background

The effects of being insufficiently active and the need to implement public policies to fight this health issue have been widely endorsed by the evidence of recent decades [1]. Research around the world reports that at least 50% of daily activities during the waking hours are performed while sitting among the adult population [2, 3]. Sedentary behaviour (SB) is defined by activities with an energy expenditure < 1.5 metabolic equivalents when seated, reclined or lying down while awake [4, 5]. Some studies indicate that the time spent in SB is detrimental to health since it has been associated with biological disease markers [3, 6, 7], and mental health outcomes [8], among others. Nevertheless, some deleterious effects on health could be reversed if people become physically active [9–11].

Given the growing body of evidence about SB, efforts are underway to design and improve measuring tools to gain a better picture of this behaviour in society [12–14]. In the last decade there has been increasing use of objective instruments, such as accelerometers, to measure physical activity (PA) and SB, as they provide totals of accumulated time at different intensities of PA and its distribution over time [15–17]. Yet despite their numerous advantages, these devices do not report contextual information about behaviour [15].

Self-reporting methods can complement the objective measurement methods or provide a better alternative when the cost of implementation is an issue [18, 19]. Therefore, the use of self-reporting instruments that can, in addition to contributing the total time, provide information on the different domains is necessary to be able to produce more specific contextual information for decision-making.

Among the self-reporting methods, questionnaires are the most common way to assess SB in population studies [20]. However, information about valid and specific self-reporting instruments in Spanish to measure SB is limited [15]. This is highly relevant as most Latin American countries have Spanish as official language, and it is the second most spoken language in the world after Chinese [21]. Also, few Latin American countries have available country-level data in sitting time or SB [22]. Some global instruments in Spanish have been used to measure PA and SB [15, 22], still, they do not provide contextual information because they only include one question about the total time SB in a typical day [23, 24].

Multi-item questionnaires for measuring SB may provide advantages compared with single-item instruments in terms of overall accuracy and the ability of capturing SB of different types and domains [15]. Some multi-item questionnaires base their responses on the participant’s activities in the last week, last day, or last weekend to facilitate recall [25, 26]. In this line, Clark and colleagues developed and validated the instrument called the *Past-day Adults’ Sedentary Time* (PAST) questionnaire [26]. The PAST asks for recall of the last day to obtain the total hours in SB in 7 contexts or domains (work, transportation, television, computers/Internet/video games, reading, hobbies, other purposes). With this format, the PAST has reported greater accuracy than the usual methods that incorporate the concept of a “typical day” such as global PA questionnaires [26, 27]. The PAST have shown similar accuracy properties between the original tool validated in a sample of breast cancer survivors compared with the version tested in university staff and students [27]. This suggests that the PAST may be applied in different populations or contexts. However, its application in other regions like South America, currently is not possible due to the PAST was originally developed and validated in English as well as some examples used in the questions may not be culturally relevant for the Latin American region.

Given the importance of considering SB an aspect to be measured in population studies, the validation of an instrument in Spanish that contributes contextual information and total SB times is necessary [22]. To fill this gap, while also providing a better opportunity for improving accuracy, a multi-item tool like the PAST offers advantages for its use in research. For this reason, the general aim of this study was to culturally adapt, translate and validate the 7-item PAST questionnaire in a population of Chilean workers.

## Methods

This study involved the translation, adaptation, and validation of the PAST questionnaire, using a cross-sectional design. The participants from the study signed an informed consent that was approved by the Scientific Ethics Committee of the Universidad de La Frontera, Temuco, Chile (n 103/2014).

### PAST questionnaire

The PAST questionnaire was developed by Clark et al. to provide contextual information about SB by domains [26]. This instrument has seven items and can be completed in approximately 20 min. The seven domains of SB are work, transportation, television, computers/Internet/video games (excluding work), reading (excluding work), hobbies, other purposes (not reported previously in the other items). Respondents are requested to provide the time in hours and minutes spent sitting or lying in those domains. The continuous time reported in the seven items is added to estimate total sedentary time.

These are some examples of the questions included in the original PAST questionnaire [26]: 1) “*How long were you sitting at your workplace or working from home yesterday, including during meal and snack breaks?”;* 2) “*Thinking again of yesterday, please estimate the total time that you spent sitting to travel from one place to another. Please include sitting and waiting for transport. Do not include any time you were standing up while travelling or waiting*”; 3) “*Please estimate the total time you spent sitting or lying down to watch TV or DVDs or play games on the TV, such as play station yesterday? This includes if you watch TV in bed*”; The other questions may be accesed from the original questionnaire in English in the following link http://links.lww.com/MSS/A252.

The PAST has demonstrated moderate to good validity in Australian adult population (range = 0.57–0.78) compared with ActivPAL [26, 27]. It has also reported an average difference in total SB of 5–25 min per day vs the ActivPAL (0.8–5.2%) [26, 27], which is less than other methods (4–5 h) [23, 28].

### Cultural and linguistic adaptation of the questionnaire

The PAST questionnaire was adapted culturally and translated from English to Spanish according to standardised criteria [29]. Stage 1 of translation from English to Spanish was done by an informed translator (t1) and one not informed (t2). Then in a meeting (stage 2) versions t1 and t2 were synthesised, and any discrepancies resolved by the research team. This new version was back-translated to English (stage 3) from Spanish by native English translators (bt1 and bt2). The research group reviewed the translation to English (bt1 and bt2) and compared these results to the synthesis in Spanish (t1 and t2) to develop a preliminary version (Stage 4). This version was tested (stage 5) in a small sample of adults (*n* = 40, university staff and students), but not included in the sample for the validation process. General observations of the participants from the test were made for the review, analysis, and final design of the version in Spanish (stage 6). Regarding the cultural adaptation, examples in some of the questions were added to clarify to what they referred, without altering the understandability of the items. As a result, we included the following examples or clarifications at the end of the questions for some domains: 1) Item 4, Transport: *“if you cycle for transportation, do not include that time either. If your job includes sitting in a vehicle, do not include that time (e.g., taxi driver).”*; 2) Item 5, Watching television: *“Only include the time when this was your main activity (e.g., if you watched TV and used your computer or ate, report only one).”;* and 3) Item 7, Reading: *“Remember, do not include reading time that has been part of any previously reported.”* The final version of the PAST in Spanish is publicly available in this link 10.6084/m9.figshare.13539056.v1.

### Reliability and validity

For this stage, workers over 18 years were invited to participate through printed news, telephone calls, and mailings to workplaces in Temuco, Chile. The only exclusion criterion being the use of a wheelchair. The validation sample included participants from different working sectors: public transport, schools, childcare, healthcare, higher education, sales and administration, and others.

The participants were visited twice in their place of work. The first visit was arranged to collect sociodemographic characteristics (age, weight (Seca 803, Germany), height (Seca 213, Germany), educational level, occupation, and self-perceived health), answer the PAST questionnaire and deliver an ActivPAL 3™μ accelerometer (ActivPAL, Pal Technologies Ltd., UK) (T1).

To assess the reliability of the PAST, the participants responded to the questionnaire during the first visit (T1), and a second visit (T2) that was completed a week later. The concurrent validity of the PAST was tested versus the measurement from the ActivPAL. Each participant received oral and written instructions on the wear of the ActivPAL for the following 7 days. Still, they were asked to perform their usual activities during the week of monitoring. The participants were also asked to complete a diary of accelerometer use and hours of sleep for the following 7 days, where they detailed their work schedules. The reference instrument for the validation was the ActivPAL. The ActivPAL is small (23.5 × 43 × 5 mm) and light (10 g). This device presents minimal differences with direct observation (0.1–2.8%) [13, 30]. Also, it is more sensitive to changes than other objective measuring instruments, like the ActiGraph GT3X [31, 32]. The device was sealed with a latex protector and adhered to the participant’s front right thigh with a transparent and hypoallergenic tape (Tegaderm™ Roll, 3M™) to provide a waterproof barrier. This allows its continuous 24-h wear for 7 days without needing to remove it for activities like showering or sleeping, for example.

After a week, the second visit was made, where the participant’s ActivPAL was removed, completed the PAST, and they were asked if they had difficulties or discomfort using the accelerometer (T2). Also, the participants were asked to report their most preferred instrument between the PAST and ActivPAL to measure SB in a future study.

### Accelerometer data reduction

The ActivPALs were started up and downloaded with ActivPal™ Professional Software, v7.2.32 Research Edition (Pal Technologies Ltd., UK). The data were extracted from the ActivPAL events file, which includes continuous intervals in seconds of activity, for subsequent integration with the participant’s diary. A semiautomatic filter was used to extract the time of use and the hours in which the participant was awake [33]. To be considered valid, the participant’s data had to include at least 10 h of daily use for 5 days of the week [34–36]. Average times were calculated for seated/lying (i.e., SB), standing, stepping, and total transitions from sitting to standing for an average day, the previous day, and at work. Also, relative estimates for time spent in SB at work were calculated for both the ActivPAL and PAST. To be able to compare the data reported with the PAST, the SB time of the previous day was used.

### Statistical analysis

To describe the study sample, measures of central tendency were used for continuous variables and percentages for the categorical variables. Comparisons of continuous variables between sexes were performed with T-test or Mann–Whitney U test, while categorical variables were compared with chi-square and proportion tests. For the analysis, stratifications by sex were performed due to women and men may accumulate SB differently throughout the day, thus affecting the self-reported estimations in the different contexts [37], including work [38]. Test-retest reliability of the PAST instrument was assessed by comparing the results obtained in T1 and T2 with the intraclass correlation coefficient (ICC). The criteria for ICC values classification were: < 0.50 as poor, 0.50- < 0.75 as moderate, 0.75- < 0.90 as good, and ≥ 0.90 was considered as excellent [39]. The concurrent validity between the PAST questionnaire and the ActivPAL was evaluated with Pearson’s (total time in SB and at work) and Spearman’s (SB percentage from work) correlations and the Bland-Altman method to estimate mean bias and limits of agreement (LoA) [40]. The criteria to evaluate the correlation between sitting time estimated by the PAST and the reference were: 0.00–0.19 as very weak, 0.20–0.39 as weak, 0.40–0.59 as moderate, 0.60–0.79 as strong, and 0.80–1.0 as very strong [41]. Paired T-test and Wilcoxon signed-rank test were used to compare total SB estimates between the PAST and ActivPAL.

The Kappa method was used to determine the agreement between tertiles, quartiles, and quintiles of SB as classified by the PAST and the ActivPAL. The following criteria were used to assess the agreement: 0.01–0.20 as none to poor, 0.21–0.40 as low, 0.41–0.60 as moderate, 0.61–0.80 as substantial, and 0.81–1.00 as almost perfect agreement [42]. All the calculations were done with a 95% confidence level. All data cleaning and analysis were done with Stata, version 15 (StataCorp. College Station, TX, USA).

## Results

One hundred one participants were recruited for the study, of which 11 did not complete the 7 days of follow-up (lost ActivPAL: 1, forgot to use ActivPAL: 9, skin irritation: 1). Also, some participants did not provided enough valid data for at least 5 days (*n* = 13). Finally, 77 adults (41 men; age = 38.5 ± 1.36 years; 67.7% overweight/obese) had valid data to complete the analyses since only participants who provided data corresponding to workdays were included (Table 1). Of all the participants with valid data, 20.2% reported itchy skin, 6.7% reported irritation, and 3.4% reported discomfort when using the ActivPAL. No differences were found as the most preferred instrument between the PAST (50.7%) and ActivPAL (49.3%, *p* = 0.493) by the participants.

**Table 1:** Sample characteristics

Variable	Total (*n* = 77)	Men (*n* = 41)	Women (*n* = 36)	*p*
Age (average, SD) years	38.5	38.9	38.1	0.78
	1.36	1.85	2.03	
Nutritional status (%)				
Underweight	1.1	–	2.3	< 0.001
Normal	31.1	10.9	52.3	
Overweight	44.4	56.5	31.8	
Obese	23.3	32.6	13.6	
Marital status (% married/de facto)	40.0	52.2	27.3	0.016
Educational level (%)				
Complete secondary education	18.9	30.4	6.8	0.017
University degree	50.0	43.5	56.8	
Graduate studies	31.1	26.1	36.4	
Occupation (%)				
Administration	10.4	17.0	2.8	< 0.001
Education	50.6	34.2	69.4	
Health	16.9	9.8	25.0	
Transportation (driver)	9.1	17.0	0.0	
Others	13.0	22.0	2.8	
Self-perceived health (%)				
Poor	16.7	17.4	15.9	0.675
Good	52.2	47.8	56.8	
Very good/Excellent	31.1	34.8	27.3	

Table 2 shows the device-measured movement behaviours in the sample of Chilean working adults. The total average time in SB measured with the ActivPAL was 553.7 ± 128.63 min/day with men accumulating more time in SB than women (588.8 vs 513.7 min/day, *p* = 0.01). On average, men were more sedentary than women (305.2 min/day vs 229.3 min/day, *p* = 0.01) during working hours. Standing time was larger in women than men on both an average day and the previous measured day. More details are shown in Table 2.

**Table 2:** Device-measured movement behaviours in the sample of Chilean working adults

Variable	Total (*n* = 77)	Men (*n* = 41)	Women (*n* = 36)	*p*
Device-measured movement behaviours on an average day				
Waking time (average, SD) min/day	979.0	988.8	967.9	0.248
	78.50	75.33	81.60	
Sedentary time (average, SD) min/day	553.7	588.8	513.7	0.01
	128.63	133.85	111.18	
Standing time (average, SD) min/day	299.8	274.6	328.4	0.004
	83.95	78.69	81.50	
Stepping time (average, SD) min/day	125.6	125.4	125.9	0.960
	44.39	52.97	32.75	
Transitions to standing (average, SD)	74.5	71.4	78.0	0.301
	27.96	30.31	24.97	
Device-measured movement behaviour on an average workday				
Working time (mean, SD) min/day	524.8	550.1	495.9	0.044
	118.21	138.20	83.08	
Sedentary time (mean, SD) min/day	269.7	305.2	229.3	0.014
	136.39	155.87	97.31	
Standing time (mean, SD) min/day	186.1	172.7	201.5	0.092
	74.64	75.46	71.67	
Stepping time (mean, SD) min/day	73.8	74.8	72.6	0.799
	37.73	42.95	31.31	
Transitions to standing (mean, SD)	38.3	38.0	38.7	0.861
	18.82	20.80	16.57	
Device-measured movement behaviours on the previous day				
Waking time (average, SD) min/day	1023.7	1026.6	1020.4	0.823
	118.80	77.82	154.0	
Sedentary time (average, SD) min/day	590.9	625.6	551.3	0.086
	189.14	196.51	174.70	
Standing time (average, SD) min/day	310.1	281.4	342.7	0.034
	127.23	133.69	112.56	
Stepping time (average, SD) min/day	122.7	119.5	126.4	0.618
	60.07	72.30	42.83	
Transitions to standing (average, SD)	76.4	73.2	79.9	0.378
	33.29	38.37	26.45	
Device-measured movement behaviours on the previous day at work				
Working time (mean, SD) min/day	532.3	559.4	497.0	0.032
	120.62	137.4	84.20	
Sedentary time (mean, SD) min/day	285.3	316.6	245.8	0.076
	165.96	191.16	119.07	
Standing time (mean, SD) min/day	208.6	187.5	233.2	0.119
	126.94	114.31	137.90	
Stepping time (mean, SD) min/day	68.5	68.6	68.4	0.985
	41.86	48.84	31.35	
Transitions to standing (mean, SD)	40.9	41.7	39.8	0.763
	25.52	27.67	22.83	

When using the PAST questionnaire (Table 3), the average time in SB was 645.8 ± 238.31 min/day. The activities adding up more sedentary time, aside from work, were watching television (96.1 ± 73.56 min/day) and using a computer or electronic devices (79.7 ± 89.10 min/day). No differences were observed between men and women in any of the domains evaluated by the PAST.

**Table 3:** Time spent in sedentary behaviours as measured with the Spanish version of Past-day Adults’ Sedentary Time (PAST) questionnaire in Chilean working adults

PAST questionnaire Domains	Total (*n* = 77)	Men (*n* = 41)	Women (*n* = 36)	*p*
Total time (mean, SD) min/day	645.8 (238.31)	653.9 (260.62)	636.6 (213.37)	0.75
Work (mean, SD) min/day	303.5 (186.82)	336.3 (215.77)	266.1 (140.99)	0.10
Transportation (mean, SD) min/day	56.2 (55.32)	47.4 (49.03)	66.2 (60.88)	0.14
Television (mean, SD) min/day	96.1 (73.56)	95.5 (80.35)	96.8 (66.11)	0.94
Computer/electronic device (mean, SD) min/day	79.7 (89.10)	78.4 (99.72)	81.1 (76.60)	0.90
Reading (mean, SD) min/day	35.7 (54.45)	31.6 (55.28)	40.4 (53.88)	0.48
Pastimes (mean, SD) min/day	16.1 (35.21)	9.3 (27.87)	23.9 (41.08)	0.07
Others (mean, SD) min/day	58.5 (56.40)	55.4 (56.74)	62.1 (56.59)	0.60

*Abbreviations*: *PAST* Past-day Adults’ Sedentary Time, *SB* sedentary behaviour, *SD* standard deviation

### Reliability

The PAST questionnaire showed moderate reliability for estimating total SB in the total sample (Table 4), while the reliability was good in men and excellent in women. The reliability was moderate when assessing time spent sedentary at work.

**Table 4:** Psychometric properties of the Past-day Adults’ Sedentary Time (PAST) questionnaire for measuring sedentary behaviour in a sample of Chilean working adults with the AP as reference standard

	Total (*n* = 77)		Men (*n* = 41)		Women (*n* = 36)	
	Estimate (CI 95%)	*p*	Estimate (CI 95%)	*p*	Estimate (CI 95%)	*p*
PAST test-retest reliability						
Total SB (ICC, CI 95%)	0.63 (0.35–0.92)	*< 0.011*	0.80 (0.57–1.04)	*0.017*	0.90 (0.74–1.05)	*0.017*
SB at work (ICC, CI 95%)	0.40 (0.13–0.68)	*< 0.001*	0.32 (0.00–0.73)	*0.075*	0.28 (0.00–0.72)	*0.104*
Sedentary behaviour						
Total SB (mean, min/day)						
PAST	645.8 (591.7–699.9)	*0.106*	653.9 (571.6–736.2)	*0.521*	636.6 (564.4–708.8)	*0.079*
AP	590.9 (548.0–633.8)		625.6 (563.6–687.7)		551.3 (492.2–610.4)	
SB at work (mean, min/day)						
PAST	318.4 (274.9–361.9)	*0.129*	349.7 (280.9–418.6)	*0.395*	279.0 (231.9–326.2)	*0.147*
AP	285.3 (245.7–324.8)		316.6 (254.7–378.6)		245.8 (202.1–289.5)	
SB percentage from work (% per day)						
PAST	48.8 (43.5–54.0)	*0.428*	52.8 (45.0–60.5)	*0.219*	43.8 (37.0–50.6)	*0.891*
AP	46.8 (42.0–51.6)		48.3 (41.9–54.8)		44.9 (37.3–52.4)	
Concurrent validity						
Total SB (rho, CI 95%)	0.21 (0.00–0.41)	*0.07*	0.19 (−0.12–0.50)	*0.24*	0.27 (−0.05–0.59)	*0.11*
SB at work (rho, CI 95%)	0.37 (0.14–0.60)	*0.002*	0.35 (0.04–0.66)	*0.028*	0.35 (−0.03–0.73)	*0.054*
SB percentage from work (rho, CI 95%)	0.32 (0.06–0.58)	*0.007*	0.36 (0.01–0.70)	*0.025*	0.25 (−0.08–0.58)	*0.179*
Agreement (kappa)						
Total SB						
Tertiles	0.08	*0.149*	−0.03	*0.590*	0.20	*0.048*
Quartiles	0.01	*0.424*	0.05	*0.300*	0.09	*0.704*
Quintiles	0.06	*0.434*	0.02	*0.375*	0.08	*0.533*
SB at work						
Tertiles	0.22	*0.003*	0.20	*0.031*	0.20	*0.045*
Quartiles	0.19	*0.002*	0.21	*0.012*	0.13	*0.099*
Quintiles	0.14	*0.007*	0.17	*0.012*	0.08	*0.187*
SB percentage from work						
Tertiles	0.08	*0.174*	0.11	*0.171*	0.03	*0.401*
Quartiles	0.05	*0.244*	0.04	*0.333*	0.05	*0.318*
Quintiles	0.07	*0.116*	0.11	*0.076*	0.05	*0.290*

*Abbreviations*: *AP* ActivPAL, *ICC* intraclass correlation coefficient, *PAST* Past-day Adults’ Sedentary Time, *SB* sedentary behaviour

### Concurrent validity

**Fig. 1:**
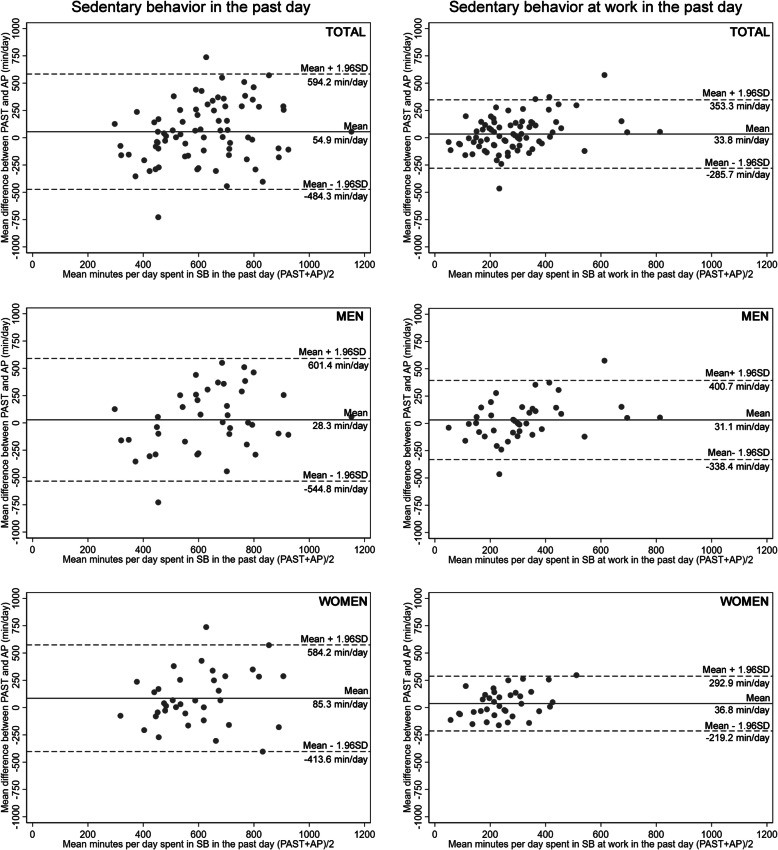
Bland-Altman difference plot for time in sedentary behaviors between PAST and ActivPAL. The solid lines represent the mean differences and the dotted lines represent the agreement limits. Abbreviations: AP: ActivPAL; PAST: Past-day Adults’ Sedentary Time questionnaire; SB: sedentary behaviour; SD: standard deviation

The SB estimates derived from both the PAST and the ActivPAL showed no differences for the total time per day, and at work, including its per cent contribution from SB to the whole day (Table 4). The PAST showed a non-significant correlation with ActivPAL for the total SB (Table 4), with an average bias of about 1 h (54.9 min/day, 95% LoA -484.3, 594.2) with the ActivPAL (Fig. 1). The women presented a mean bias of almost 1 h more than that observed in the men (85.3 [95% LoA: − 413.6, 584.2] vs 28.8 [95% LoA: − 544.8, 601.4] min/day). The agreement between the PAST and ActivPAL to classify the mean sedentary time into tertiles, quartiles, and quintiles was not significant (Table 4).

Overall, the PAST showed a weak correlation with the ActivPAL for the total sedentary time at work (Table 4). The mean bias between the PAST and ActivPAL was about half an hour per day for SB during working hours, with similar differences for men and women (Fig. 1). The agreement between the PAST and ActivPAL to classify the time spent in SB during working hours was poor to low when tertiles, quartiles and quintiles were used (Table 4).

**Fig. 2:**
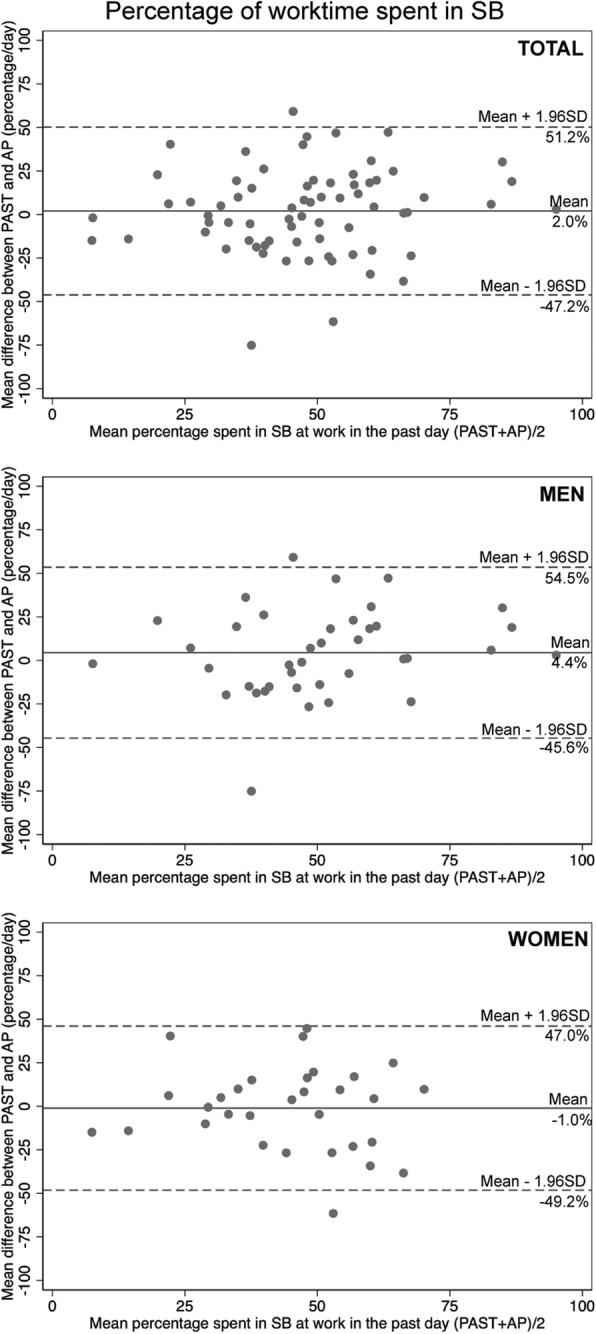
Bland-Altman difference plot for the percentage of worktime spent in sedentary behaviors between PAST and ActivPAL. The solid lines represent the mean differences and the dotted lines represent the agreement limits. Abbreviations: AP: ActivPAL; PAST: Past-day Adults’ Sedentary Time questionnaire; SB: sedentary behaviour; SD: standard deviation

When comparing percentages of work time spent sedentary between the PAST and ActivPAL, a weak correlation was observed (Table 4). The average bias was 2.0% per day between the PAST and the ActivPAL (Fig. 2). No agreement between the PAST and ActivPAL was found to classify the proportion of time spent in SB at work into percentile categories (Table 4).

## Discussion

Overall, the total device-measured sedentary time corresponded to 56.6% of the day in the entire sample (men: 59.5%, women: 53.1%), while at work, the participants were sitting for almost half of the time each day. The recorded daily time in SB measured objectively was comparable to other studies conducted on a Chilean adult population [23, 43]. The reliability of the PAST questionnaire was moderate, and its validity was weak compared with the ActivPAL to estimate SB, but only during working hours.

The reliability of the PAST showed similar results to those reported previously with the English version in Australian adult and university populations [26, 27]. However, our findings on the concurrent validity of the PAST were dissimilar to those reported previously in Australia, since, in those studies, the validity was moderate to substantial [26, 27]. These differences may be due to our research included adults with various occupations and physical activity requirements compared to the Australian participants, which had patients with cancer and people in a university context [26, 27]. Apart from comparing between sexes, we explored different grouping options using both occupations and latent class analysis based on the movement behaviours as measured with the ActivPAL, but no improvements were found in the accuracy properties of the instrument.

The mean bias between the PAST and ActivPAL for total SB was considerably lower than the observed with the single question of the GPAQ assessed in Chilean population [23]. Our findings are in line with a recent meta-analysis in which multi-item questionnaires showed smaller mean differences with accelerometers compared with single-item instruments [15]. Although the mean bias was almost an hour, the limits of agreement between the PAST and ActivPAL were wide, indicating that the estimations on the individual level are very limited. These large differences might be because some study participants over or under-reported some sedentary activities without a clear pattern, as found in several studies from a meta-analysis [15]. For example, some people may have reported time on the computer for recreational or work activities separately, while others may have included that as part of the estimation only at work. During the data collection, efforts were made orally to avoid these confusions, as well as in the written instructions. Still, each participant was free to report the times that they estimated.

As shown in a recent meta-analysis, the concurrent validity of the PAST for estimating total SB was comparable to that commonly observed in other self-report methods [15]. The findings were also similar with other instruments available in Spanish [23–25]. As shown in other studies, the PAST questionnaire performed better for both when measuring time in SB at work and comparing the proportion of their workday spent in SB [15]. The use of percentages could be a complementary approach to explore in the future as the current and previous studies have shown no differences between overall recall estimates based on proportions and those derived from devices [15]. Following this principle, it has been suggested that a visual analogue scale could be used to measure the proportion of the day spent sitting as it has shown higher precision and data loss when compared with other tools [14]. The slightly better performance of the PAST in work context could be explained due to the time in SB during the workday being less variable between days, unlike free time activities, which were also observed in the initial validation of the PAST [26].

Self-report measures are valuable tools as they provide contextual information that could be very difficult to register with accelerometers or other devices [20]. Also, when comparing prospective studies that have been used self-reported and device-measured SB, the direction of the associations with outcomes, such as mortality, are consistent [44]. Although the self-reports performance is usually poor to moderate compared with devices [15], these methods will remain as relevant tools as more interest has been placed into differentiating the influence of different sedentary domains or activities on outcomes, such as mental health [8, 45, 46]. Due to the lower cost of self-reports compared with accelerometers or inclinometers, these instruments play an important, and sometimes unique, role to conduct studies in settings where resources are limited, or research is incipient, like in Chile. Therefore, this study fills an important gap to better understand sedentary behaviours in a Latin American context.

### Strengths and limitations

This study was the first to validate the PAST questionnaire in Spanish. Although the recruitment was not probabilistic, it managed to include participants from various work environments and with a wide age range. This improves the external validity of the findings reported in this study about a working adult population. A major limitation of the study was that despite 101 participants were recruited, only 77 were included in the analyses, mainly for not wearing the accelerometer (i.e., enough data on at least 5 days). Nevertheless, there were no differences in the demographic characteristics between those included and not included in the study. This instrument only evaluates the SB from the last day, which could limit its use to estimate common SB that could be more variable, particularly in free time [47]. Although the PAST assesses SB in different domains, apart from the whole day, we were able to explore only the time spent sedentary at work. This remains an issue in the field due to the difficulty of assessing SB in different contexts [14], so the use of cameras or logs as reference standards may be useful tools to fill this gap in future studies.

## Conclusions

The PAST questionnaire showed moderate reliability, but no correlation with the ActivPAL to estimate SB from the last day. Slightly better results were obtained to evaluate both absolute and relative estimates of SB at work. Overall, sedentary time showed no difference between the PAST and the ActivPAL on an average day and at work, but the individual variability of the differences was wide. Therefore, the PAST may be useful for overall group rather than individual estimates of SB.

## Data Availability

Data can be shared based on specific requests to the corresponding author.

## Abbreviations

ICC: Intraclass correlation
IPAQ: International Physical Activity Questionnaire
GPAQ: Global Physical Activity Questionnaire
PA: Physical activity
PAST: Past-day Adults’ Sedentary Time
SB: Sedentary behavior
